# Is there a correlation between signal intensity ratio of 3T MRI and molecular subtypes of breast cancer?

**DOI:** 10.2478/raon-2026-0029

**Published:** 2026-07-04

**Authors:** Yuanping Tao, Hongyan Chao, Yue Ren, Wanjun Zheng, Yuyou Chen, Liang Du, Huanguo Li, Feng Cui

**Affiliations:** 1 Department of Radiology, Hangzhou TCM Hospital Affiliated to Zhejiang Chinese Medical University, Hangzhou, China; 2 School of Economics, Center for Economic Behavior and Decision-making (CEBD), Zhejiang University of Finance and Economics, Hangzhou, China

**Keywords:** breast cancer, signal intensity ratio, molecular subtype, MRI

## Abstract

**Background:**

The study aimed to test whether tumoral signal intensity ratio (SIR) and other indicators of 3T MRI provides information related to molecular subtypes of breast cancer.

**Patients and methods:**

Between January 2022 and August 2025, 256 patients with breast cancer underwent 3T MRI. MRI morphological features and quantitative parameters (T1 SIR, T2 SIR, mean apparent diffusion coefficient [ADC] from diffusion-weighted imaging [DWI] and tumor diameter) were compared and measured according to molecular subtypes. Logistic regression was performed to find the related MRI parameters and establish combined parameters. A receiver operating characteristic (ROC) analysis was finally performed to test the diagnostic power of multivariate logistic regression model.

**Results:**

263 lesions were considered. Triple-negative (TN) subtype exhibited the highest T2 SIR and lowest T1 SIR (p < 0.05). Mean ADC values in TN were the lowest and highest in human epidermal growth factor receptor 2 (HER2)-enriched type (p < 0.001). Tumor diameter of HER2-enriched was the smallest, and that of triple-negative subtype was the largest (p < 0.001). Independent influencing factors were higher T2 SIR (p = 0.017) and larger tumor diameter (p = 0.011) associated with triple-negative subtype, and T1 SIR (p = < 0.01) was the only quantitative parameter associated with HER2-enriched subtype. The combined parameter (mean ADC + tumor diameter) achieved a largest area under the ROC curve (AUC = 0.781) for separating luminal A subtype.

**Conclusions:**

Quantitative MRI parameters are correlated with the molecular subtypes of breast cancer. Combined parameters incorporating T1 SIR and T2 SIR exhibit potential diagnostic utility for differentiating HER2-enriched and triple-negative subtypes, respectively. T1 SIR and T2 SIR can enrich quantitative descriptors from breast MRI.

## Introduction

Magnetic resonance imaging (MRI) has very high sensitivity in detecting breast cancer, with detection rates ranging from 95% to 99%.^1^ Breast MRI is also the most accurate imaging method for evaluating tumours size, multifocality and multicentricity.^2^ The morphological and contrast-enhanced features, diffusion-weighted imaging (DWI) and T2 weight imaging (T2WI) in MRI are helpful for diagnosing lesions.^3^ In addition to the complete diagnostic protocol, simplified rapid breast MRI also has certain diagnostic accuracy and sensitivity in detecting breast cancer.^4,5^ In recent years, MRIbased quantitative metrics, including dynamic contrast-enhanced MRI (DCE-MRI) and DWI, as well as derived parameters such as T1 and T2 relaxation time and proton density (PD) image, have shown great potential in the non-invasive diagnosis and evaluation of breast cancer.^6–10^ The MRI signal intensity ratio (SIR) has been used as a quantitative measure in the other diseases.^11–14^ However, there are still few studies on the SIR of breast cancer.^15^ Therefore, we attempted to analyse the correlation between T1 SIR, T2 SIR, ADC values, lesion diameters combining with other morphological and enhanced scanning characteristics and the different molecular subtypes of breast cancer.

## Patients and methods

### Study participants

**FIGURE 1.**
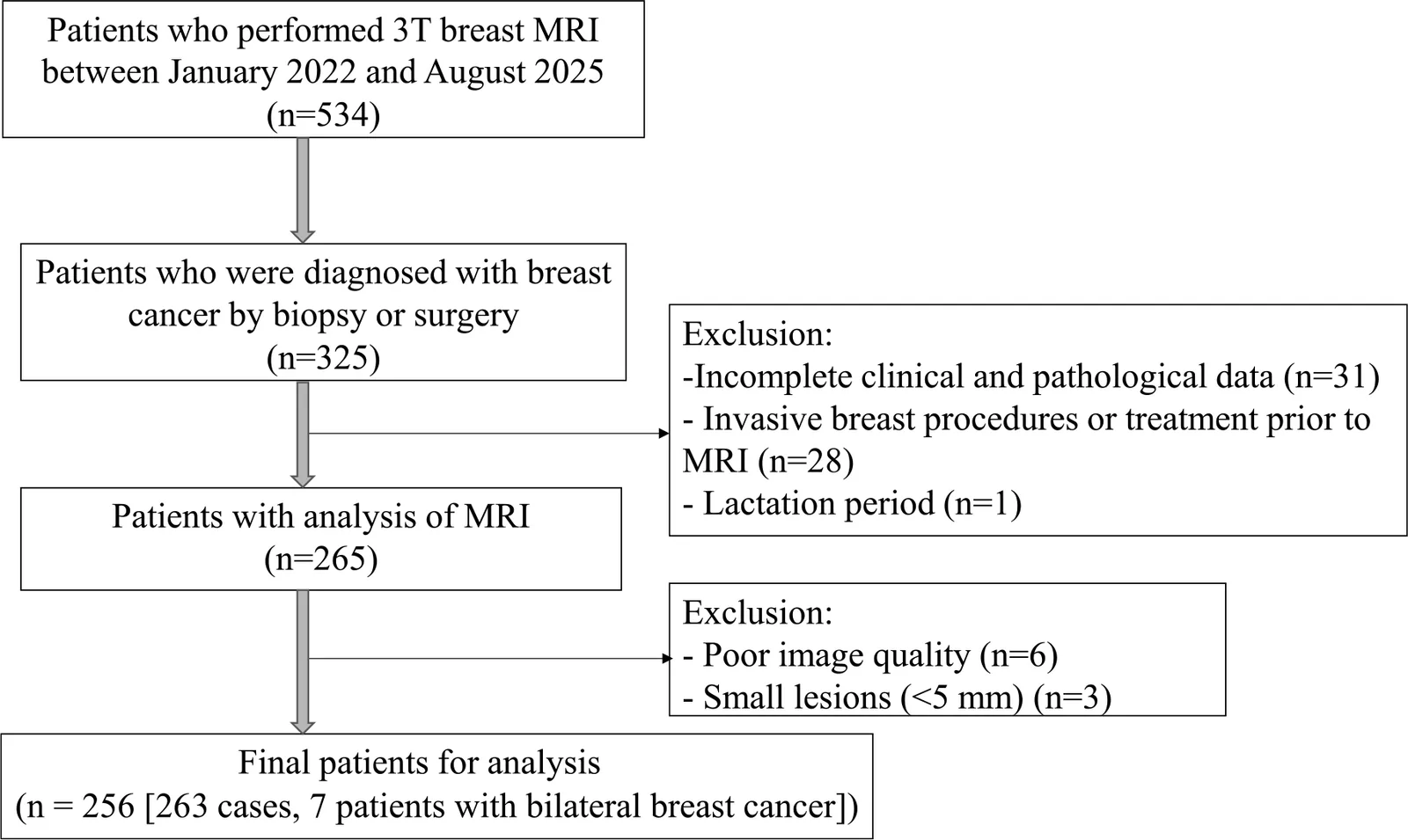
Inclusion and exclusion criteria and the data collection flowchart showing the study population.

From January 2022 to August 2025, we consecutively collected 534 patients who underwent 3T breast MRI in the radiology department of one hospital. These patients were derived from routine breast cancer screening, those with breast lesions of uncertain nature, and individuals with previously confirmed breast cancer. All patients had no MRI contraindications, and breast lesions were classified as breast imaging reporting and data system (BI-RADS) category 4A or 5 on mammography or ultrasound. A total of 332 cases of breast cancer (from 325 patients, including 7 patients with bilateral breast cancer) were pathologically diagnosed with breast cancer. This study was approved by the institutional review boards of our centre (ethics committee approval number: 202310130922000244065), and the requirement for informed consent was waived. This study complied with the principles of the Declaration of Helsinki. The inclusion criteria were as follows: (1) breast cancer confirmed by pathological assessment of the resection specimen or biopsy; (2) molecular subtype validated by immunohistochemistry (IHC) and human epidermal growth factor receptor 2 (HER2) gene amplification status validated by fluorescence in-situ hybridization (FISH); (3) breast MRI examination completed less than 1 month prior to biopsy or resection. The exclusion criteria were as follows: (1) incomplete clinical and pathological data; (2) breast-related invasive procedure (such as biopsy less than 2 weeks interference of bleeding and inflammation on imaging features) and treatment (chemotherapy, radiotherapy, or hormone therapy) before MRI examination; (3) pregnant or lactation period and (4) poor image quality or small lesion (e.g., motive artifacts, smaller than 5mm lesion). Based on the exclusion criteria, 69 patients were excluded because of incomplete clinical and pathological data (n= 31), breast-related invasive procedure and treatment before MRI examination (n = 28), lactation period (n = 1), poor image quality (n = 6) and small lesions could not be measured (n = 3). A flowchart of study population is shown in Figure 1. Thus, 256 patients with a total of 263 lesions (7 patients with bilateral lesions) of breast cancer were ultimately enrolled in this study.

### MRI protocol

3.0T MRI scanner (Discovery 750 3.0T, GE Healthcare, Milwaukee, USA) was used, with a dedicated eight-channel phased-array coil for the breast. The patient was placed in a prone position, with both arms raised, keeping the body basically parallel to the scanning bed. The two breasts were adjusted to hang naturally in the coil, with the head first. The following sequences were scanned in turn: axial T1 and T2 weighted fat-suppressed sequence, DWI, and axial T1WI dynamic enhanced scan. The scanning parameters of axial T1WI were: repetition time (TR) = 425 m s, echo time (TE) = 8.384 m s, slice thickness = 4 mm, field of view (FOV) = 320 mm × 320 mm; the scanning parameters of T2WI fat-suppressed sequence were: TR = 5762 m s, TE = 89.408 m s, slice thickness = 4 mm, FOV = 320 mm × 320 mm; the scanning parameters of DWI sequence were: TR = 2777.26 m s, TE = 60.6 m s, slice thickness = 4 mm, FOV = 320 mm × 320 mm; the scanning parameters of T1WI dynamic enhancement were: TR = 4.537 m s, TE = 1.798 m s, slice thickness = 1 mm, FOV = 320 mm × 320 mm. Plain scan was performed as a mask before injecting the contrast agent. After the plain scan, gadolinium contrast agent (Gd - DTPA, 0.1 mmol/kg, 2.0 mL/s) was injected through the elbow vein, followed by a rapid injection of 20ml of 0.9% sodium chloride. The enhanced scan started at the 23rd second, and parallel acquisition was used to continuously scan 8 times without interruption.

### Imaging analysis

Two radiologists with 10 and 11 years of experience in diagnosing breast disease evaluated all lesions based on the BI-RADS diagnostic criteria respectively, who were blinded to histopathological results. If there was any disagreement, a consensus was finally reached through negotiation. The final quantitative parameters were the mean values of two readers. The diameter of the lesion was measured during the fourth phase of enhancement and non-mass-like lesions were excluded.

#### Quantitative parameters

**FIGURE 2.**
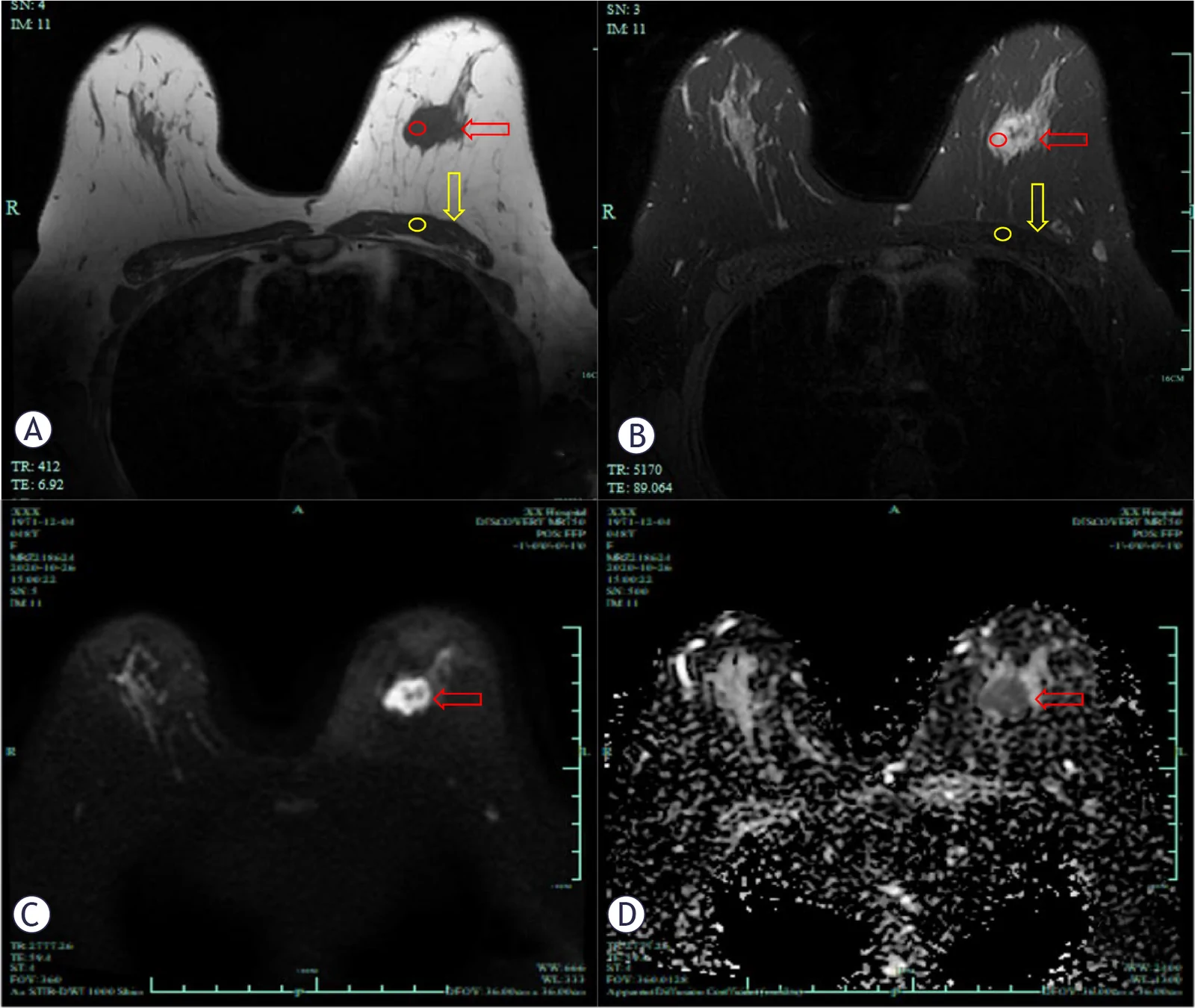
MR images of a 48 years-old patient diagnosed with luminal B subtype breast cancer. T1 weight image **(A)**, T2 fatsuppression sequence image **(B)**, DWI image **(C)** and ADC map **(D)** was shown. T1 and T2 signal intensity ratio **(A** and **B)** were calculated by proportioning the average signal intensity of the lesion (red circle) to the average signal intensity of the pectoral muscle (yellow circle). Red arrows show the lesions and yellow arrows show the pectoral muscles. ADC = apparent diffusion coefficient; DWI = diffusion-weighted imaging

For T1 and T2 signal intensity ratio (SIR) analysis, signal intensities were obtained from the lesion and the pectoral muscle, which was used as an internal reference. The SIR was calculated as the ratio of lesion-to-pectoral muscle signal intensity. Region of interests (ROIs) drawn in the lesion and pectoral muscle were of identical size. On T1 and T2 weighted axial image, ROI of the lesion was adjusted based on contrast enhancement in DCE-MRI, and non-enhancing, necrotic, and cystic areas were carefully disregarded to include only solid tumour components. ROI of the pectoral muscle was obtained at the thickest layer of the pectoralis major muscle, avoiding fatty tissue (Figure 2).

#### ADC values

ROI was drawn within the tumour lesion on the ADC map based on the DWI image with b-value of 800. The location of the ROI was adjusted according to the higher region on DWI and enhancement on DCE-MRI, and the mean ADC values were recorded for each lesion.

#### Histopathological analysis

The pathologist with 10 years of experience in breast disease, who was blinded to the results of MRI. ER and/or PR positivity was considered hormone receptor (HR)-positive, while both ER and PR negativity was considered HR-negative. A HER2 status of 0 or 1+ was considered negative, 2+ was considered ambiguous, and 3+ was considered positive. Patients with an ambiguous HER2 status were evaluated using fluorescence in situ hybridization (FISH); if HER2 gene amplification was observed, they were considered positive. The Ki-67 expression was classified as high if the staining positivity was more than 14% and low if it was equal to or less than 14%. Tumor subtypes were categorized as follows: Luminal A(HR-positive and Ki-67≤14% and HER2-negative), luminal B (HR-positive with either ki-67≥14% and/or HER2-positive), HER2-enriched (HR-negative and HER2-positive) and triple-negative (TN) (ER- and PR-negative and HER2-negative)^16^.

#### Data and statistical analysis

The normality of continuous variables was evaluated by using the Shapiro–Wilk test. Normally distributed variables are expressed as the means ± standard deviations; abnormally distributed variables are expressed as the median with the first and third interquartile range (IQR). Levels of inter-reader agreement were assessed via the intraclass correlation coefficient (ICC) test. The Kruskal-Wallis H test for continuous variables and the Chi-square test or Fisher’s exact test for categorical variables were used to evaluate the relationship between MRI findings and clinical data with molecular subtypes. Benjamini-Hochberg method was invoked to correct p values for multiple testing. Receiver operating characteristic (ROC) curve analysis was performed to evaluate the performance of the binary classifier system and was compared by DeLong tests. Univariate and multivariate logistic regression analysis were performed to screen the independent risk factors associated with one specific subtype and establish combined parameters. Variables with p < 0.1 in the univariate analysis were used in the multivariate analysis. The Stata 16.0 was used for statistical analysis. p < 0.05 was considered statistically significant.

## Results

### Clinical data of patients and MRI morphological features with different molecular subtype

All 263 cases breast cancer patients were female, including 52 (19.8%) cases of luminal A type, 135(51.3%) cases of luminal B type, 31 (11.8%) cases of HER2-enriched type and 45 (17.1%) cases of triple-negative type. For clinical characteristics, there were significant differences in axillary lymph node metastasis (ALNM) (p = 0.01) and history of hormone use (p = 0.038) for four molecular subtypes, and no statistical differences in age, uterine fibroid, menopause and fertility. For MRI morphological features, there were slightly significant differences in tumor morphology (p = 0.053), and no statistical differences in margin and dynamic curve (Table 1).

**TABLE 1.** Clinical characteristics of patients and MRI morphological features for different molecular subtypes

Parameters	Luminal A (n = 52)	Luminal B (n = 135)	HER2-enriched (n = 31)	TN (n = 45)	p
ALNM					**0.010**
Yes	9 (17.3)	58 (43.0)	12 (38.7)	14 (31.1)	
No	43 (82.7)	77 (57.0)	19 (61.3)	31 (68.9)	
History of hormone use					**0.038**
Yes	3 (5.8)	2 (1.5)	3 (9.7)	0 (0.0)	
No	49 (94.2)	133 (98.5)	28 (90.3)	45 (100.0)	
Tumor morphology					0.053
Mass	43 (82.7)	114 (84.4)	20 (64.5)	39 (86.7)	
Non-mass	9 (17.3)	21 (15.6)	11 (35.5)	6 (13.3)	
Margin					0.206
Circumscribed	21 (40.4)	39 (28.9)	13 (41.9)	19 (42.2)	
Not-circumscribed	31 (59.6)	96 (71.1)	18 (58.1)	26 (57.8)	
Dynamic curve curve					0.248
type I	8 (15.4)	13 (9.6)	4 (12.9)	7 (15.6)	
type II	9 (17.3)	30 (22.2)	12 (38.7)	8 (17.8)	
type III	35 (67.3)	92 (68.1)	15 (48.4)	30 (66.7)	

1 Categorical variables are numbers with percentages in parentheses. Bolded text indicates statistical significance (p < 0.05).

1 ALNM = axillary lymph node metastasis; HER2 = human epidermal growth factor receptor 2; TN = triple-negative

### Interobserver agreement of quantitative parameters

T1 SIR, T2 SIR, mean ADC values and tumor diameter showed excellent interobserver agreements with ICC > 0.8 (Table 2).

**TABLE 2.** Intraclass correlation coefficient (ICC) analysis between the two readers

Parameters	ICC (95%CI)
T1 SIR	0.970 (0.961–0.976)
T2 SIR	0.967 (0.957–0.974)
Mean ADC (×10^−3^ mm^2^/s)	0.898 (0.871–0.919)
Tumor diameter (cm)	0.923 (0.900–0.940)

1 ADC = apparent diffusion coefficient; T1 SIR = T1 signal intensity ratio; T2 SIR = T2 signal intensity ratio

### Comparison of MRI quantitative parameters for molecular subtypes

Comparison of MRI quantitative parameters according to molecular subtypes are summarized in Table 3. T1 SIR was the lowest in triple-negative type and highest in HER2-enriched type, with statistically significant difference (p = 0.026). T2 SIR was the lowest in luminal A type and the highest in triple-negative type, with significant statistical difference (p = 0.009). Mean ADC values had significant difference in four molecular subtypes (p < 0.001), in triple-negative type were the lowest and highest in HER2-enriched type. Tumor diameter of HER2-enriched type was the smallest, and that of triple-negative type was the largest, with significant statistical difference (p < 0.001).

**TABLE 3.** MRI quantitative parameters for molecular subtypes of breast cancer

Parameters	Luminal A (n = 52)	Luminal B (n = 135)	HER2-enriched (n = 31)	TNBC (n = 45)	p
T1 SIR	1.318 (1.179–1.630)	1.366 (1.209–1.620)	1.515 (1.268–1.788)	1.254 (1.141–1.401)	**0.026**
T2 SIR	3.137±1.156	3.163±1.186	3.280±1.289	3.689±1.093	**0.009**
Mean ADC (×10^−3^ mm^2^/s)	1.037±0.291	0.912±0.211	1.102±0.297	0.900±0.164	**0.000**
Tumor diameter (cm)	1.200 (0.887–1.762)	1.900 (1.325–2.725)	1.150 (0.600–2.750)	2.550 (1.700–3.750)	**0.000**

1 Normally distributed variables are expressed as the mean ± SD, and abnormally distributed variables are expressed as the median with the first and third interquartile range (IQR). Bolded text indicates statistical significance (p < 0.05).

1 ADC = apparent diffusion coefficient; HER2 = human epidermal growth factor receptor 2; T1 SIR = T1 signal intensity ratio; T2 SIR = T2 signal intensity ratio; TN = triple-negative

### Univariate and multivariate logistic regression analysis

In the univariate regression analysis, mean ADC values (OR = 4.259, p = 0.022), tumor diameter (OR = 0.593, p = 0.016) and ALNM (OR = 0.316, p = 0.012) were associated with luminal A. Mean ADC values (OR = 0.176, p = 0.012) and ALNM (OR =2.002, p = 0.018) were associated with luminal B. T1 SIR (OR = 3.277, p < 0.01), mean ADC values (OR = 7.815, p = 0.002) and non-mass (OR = 0.334, p = 0.036) were associated with HER2-enriched. T2 SIR (OR = 1.384, p = 0.02) and tumor diameter (OR = 1.256, p = 0.04) were associated with TN.

In the multivariate regression analysis, mean ADC (OR = 3.121; p = 0.041) and tumor diameter (OR = 0.652; p = 0.019) were associated with luminal A. Mean ADC (OR = 0.193; p = 0.005) and ALNM (OR = 1.908; p = 0.016) were associated with luminal B; T1 SIR (OR = 2.907; p = 0.002), non-mass (OR = 0.373, p = 0.028) and mean ADC (OR = 3.732; p = 0.069) were associated with HER2-enriched. T2 SIR (OR = 1.395; p = 0.017) and tumor diameter (OR = 1.245; p = 0.011) were associated with TN.

### Parameters for discriminating molecular subtypes

Detailed results of ROC analysis of MRI quantitative parameters for separating one subtype from all other subtypes are summarized in Table 4. To distinguish luminal A and TN from other subtypes respectively, tumor diameter achieved the largest area under the ROC curves (AUC = 0.768, p = 0.016; AUC = 0.706, p = 0.04). Mean ADC showed a largest AUC (AUC = 0.713, p =0.002) in the differentiation between HER2-enriched and other subtypes. The other quantitative parameters only showed poor discrimination or no significant difference.

**TABLE 4.** ROC analysis of MRI quantitative parameters for separating one subtype from all other subtypes

Parameters	AUC	Cutoff value	Sensitivity (%)	Specificity (%)	p*
Luminal A vs. all others					
T1 SIR	0.506	≤ 1.270	46.15 (40.1, 52.2)	61.61 (55.7, 67.5)	0.640
T2 SIR	0.550	≤ 3.000	59.62 (53.7, 65.6)	54.50 (48.5, 60.5)	0.397
Mean ADC (×10^−3^ mm^2^/s)	0.612	> 0.980	46.15 (40.3, 52.0)	70.62 (65.3, 76.0)	**0.022**
Tumor diameter(cm)	0.768	≤l.900	80.00 (73.1, 86.9)	64.2 (57.0, 71.4)	**0.016**
Luminal B vs. all others					
T1 SIR	0.510	>1.291	62.96 (56.2, 69.7)	48.44 (41.7, 55.2)	0.181
T2 SIR	0.563	≤ 3.298	65.93 (59.6, 72.3)	45.31 (38.6, 52.0)	0.169
Mean ADC (×10^−3^ mm^2^/s)	0.624	≤ 0.978	77.78 (71.6, 84.0)	44.53 (37.8, 51.3)	**0.012**
Tumor diameter(cm)	0.540	>1.550	78.63 (71.7, 85.6)	35.58 (28.2, 42.9)	0.829
HER2-enriched vs. all others					
T1 SIR	0.631	> 1.503	58.06 (50.3, 65.8)	69.40 (61.9, 76.9)	**0.000**
T2 SIR	0.517	> 2.815	67.74 (60.0, 75.5)	44.40 (36.6, 52.2)	0.927
Mean ADC (×10^−3^ mm^2^/s)	0.713	> 0.903	87.10 (81.5, 92.7)	53.02 (45.1, 60.9)	**0.002**
Tumor diameter(cm)	0.543	> 2.190	60.00 (52.4, 67.6)	60.20 (52.6, 67.8)	0.697
TN vs. all others					
T1 SIR	0.608	≤ 1.286	64.44 (58.6, 70.2)	62.84 (57.0, 68.7)	0.176
T2 SIR	0.655	>3.157	68.89 (61.8, 76.0)	59.63 (51.9, 67.4)	**0.020**
Mean ADC (×10^−3^ mm^2^/s)	0.562	≤ 0.869	51.11 (44.1, 58.1)	62.39 (54.9, 69.9)	0.087
Tumor diameter(cm)	0.706	>2.050	74.36(65.3, 83.4)	57.69 (49.3, 66.1)	**0.040**

* Corrected p values by Benjamini-Hochberg method. Data are values with 95% confidence interval in parentheses. Bolded text indicates statistical significance (p < 0.05).

1 ADC = apparent diffusion coefficient; AUC = area under the ROC curve; HER2 = human epidermal growth factor receptor 2; ROC = receiver operating characteristic; T1 SIR = T1 signal intensity ratio; T2 SIR = T2 signal intensity ratio; TN = triple-negative

**FIGURE 3.**
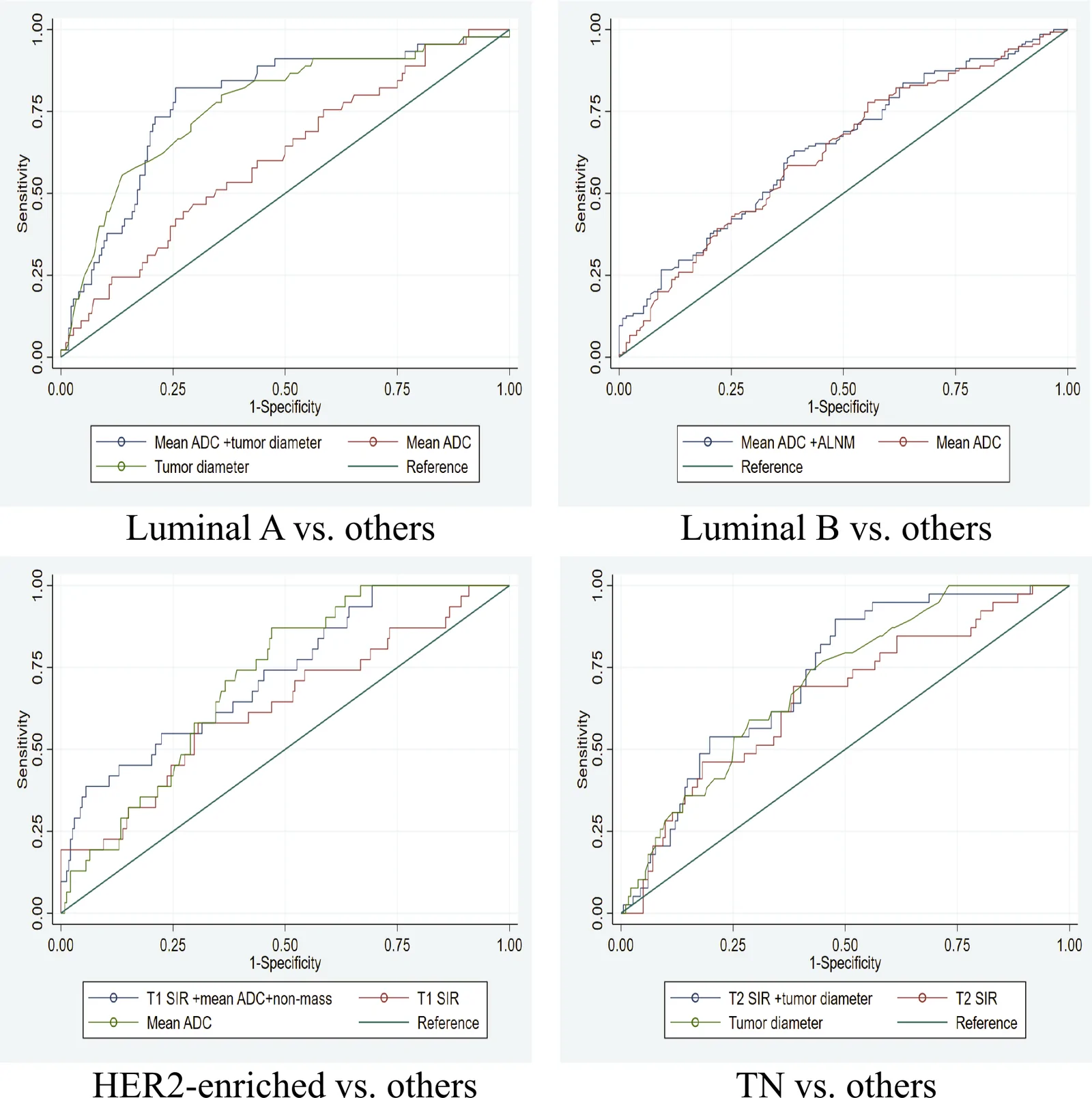
The comparison of receiver operating characteristic (ROC) curve of single and combined parameters for distinguishing one subtype from all other subtypes. ADC = apparent diffusion coefficient; ALNM = axillary lymph node metastasis; TN: triple-negative; T1 SIR = T1 signal intensity ratio; T2 SIR = T2 signal intensity ratio

MRI parameters associated with each molecular subtype in multivariate logistic regression analysis were used to establish combined parameters. The comparison of ROC curves of single and combined parameters was shown in Figure 3. The AUCs of combined parameters (Table 5) were higher than any single quantitative parameter (Table 4). Delong tests showed the diagnostic efficiency of combined parameters and single quantitative parameters were no significant difference. For separating luminal A subtype from others, the combined parameter (mean ADC+ tumor diameter) achieved a largest AUC (AUC = 0.781).

**TABLE 5.** ROC analysis of combined parameters for separating one subtype from all other subtypes

	AUC	Sensitivity (%)	Specificity (%)	p
Luminal A vs. all others				
Mean ADC+ tumor diameter	0.781	82.22 (76.57, 87.87)	74.43 (68.99, 79.87)	< 0.001
Luminal B vs. all others				
Mean ADC+ALNM	0.639	62.96 (57.12, 68.80)	60.94 (55.04, 66.84)	< 0.001
HER2-enriched vs. all others				
T1 SIR + mean ADC+ non-mass	0.728	38.71(21.56, 55.86)	94.4(91.44, 97.36)	< 0.001
TN vs. all others				
T2SIR+ tumor diameter	0.724	89.74 (85.64, 93.84)	52.20 (45.68, 58.72)	0.002

1 Bolded text indicates statistical significance (p < 0.05). Data are values with 95% confidence interval in parentheses.

1 ADC = apparent diffusion coefficient; ALNM = axillary lymph node metastasis; AUC = area under the ROC curve; HER2 = human epidermal growth factor receptor 2; TN = triple-negative; T1 SIR = T1 signal intensity ratio; T2 SIR = T2 signal intensity ratio

## Discussion

Breast cancer is a highly heterogeneous disease, and the 2017 edition of the St. Gallen Consensus divided breast cancer into 4 molecular pathological subtypes based on its gene expression profile, and stated that the four subtypes of breast cancer require different treatments.^16^ These subtypes differ significantly in clinical behaviour, prognosis, and response to treatment. Other researchers used the above four subtypes of classification^17–19^, thus we also categorized breast cancer into four subtypes in our study.

Histopathological analysis is currently the gold standard for diagnosing molecular subtypes of breast cancer in clinical practice. However, pathologists only perform hot-spot studies on surgical specimens and do not routinely evaluate all areas of the tumor. Furthermore, this is an invasive examination that may lead to complications (hematoma, inflammation, oedema), and due to the longitudinal heterogeneity of tumour volume, there may be discordance in biomarker status (ER, PR, HER2) between biopsy and surgical specimen.^20^ In contrast, with MRI, a minimally invasive procedure, evaluating all areas of the tumor and repeating these measurements is significantly easier and more advantageous than in pathology practice. MRI especially advanced quantitative MRI techniques as highly sensitive non-invasive imaging technology, can provide overall information on tumour morphology, angiogenesis, cell density and microstructure^21,22^, compensating for the limitations of biopsy or histopathological examination in tumour heterogeneity. Nevertheless, these quantitative parameters require additional image post-processing, radiomics feature extraction, selection, and model construction. The quantitative parameters in our study can be directly measured from the scan images without needing additional image post-processing, making the process simple, quick, and conducive to widespread clinical application.

The present study attempts to compare multiple quantitative parameters including (T1 SIR, T2 SIR, mean ADC values and tumor diameter) of breast cancer for discriminating molecular subtypes. We found triple-negative type had the lowest T1 SIR and highest T2 SIR, similar with some studies.^23,24^ Triple-negative breast cancer exhibits higher levels of VEGF compared to non-triple-negative type, which promotes angiogenesis, leading to increased microvascular density, indicating lower T1 signal intensity and higher T2 signal intensity. Moreover, the higher angiogenesis levels in triple-negative breast cancer may lead to local tissue oedema, further decreasing T1 SIR and increasing T2 SIR.^15,25^ Furthermore, the T2 SIR in luminal A type were the lowest in our study. This aligns with some findings. Luminal A breast cancer was related homogeneous iso/hypointense features on T2W^26^, compared to heterogeneous hypointense ones, exhibited lower proliferation, correlating with better prognosis.^27^

In our study, we also revealed that mean ADC values were the lowest in triple-negative and highest in HER2-enriched type, consistent with some studies.^28,29^ In contrast, another study indicated that ER or PR-positive tumors had lower mean ADC values.^30^ Variations in mean ADC values or diffusion restriction may result from differences in luminal A proportions across studies and the changing thresholds distinguishing luminal B from luminal A subtypes. Another contributing factor may be the inherent heterogeneity within luminal A breast cancer.

Additionally, we observed that tumor diameters in luminal A and luminal B type were smaller than triple-negative, similar with previous findings.^19^ This highlights the faster growth and invasive capability of triple-negative breast cancer compared to the relatively slow-growing luminal A and luminal B subtype.

HER2-enriched subtype had a higher proportion of non-mass lesions (35.5%) and hormone use history (9.7%) in our cohort. This observation is comparable with the study of Navarro *et al*.^31^, which reported frequent non-mass enhancement in HER2-positive breast cancer. This supports the theory of intraductal development in this subtype. Kazama T *et al*.^32^ and Coskun B A *et al*.^33^ also revealed that HER2-enriched type is closely related to high hormone level.

Our study quantified the subjective signal intensity as T1 SIR and T2 SIR and found that independent influencing factors were higher T2 SIR and larger tumor diameter associated with triplenegative subtype, and T1 SIR was the only quantitative parameter associated with HER2-enriched subtype. The combined parameters (mean ADC + tumor diameter) achieved a largest AUC (AUC = 0.781) for separating luminal A subtype. The result is similar with Chen *et al*.^34^, the AUC of combined model (mean kurtosis value + perfusion fraction + pseudo diffusion coefficient) was 0.785, and higher than that of another study combining the T2, mean ADC and tumor volume parameters to distinguish luminal A subtype (AUC= 0.765).^3^ A recent study regarding the identification of luminal A/B breast cancer subtypes^35^ demonstrated that the hybrid model significantly outperformed the individual approaches, achieving an excellent AUC (0.921) and the most important contributors were radiomic features related to shape and texture and high-level deep features.

Breast fibroglandular tissue content varies in individuals. Identification of suitable fibroglandular tissue for T1 and T2 signal measurement is challenging in extremely fatty breasts. So, the patient’s own pectoral muscle was selected as the internal control for T1 and T2 SIR.^15^ Interindividual variation in the pectoralis muscle among patients may be associated with sex, body habitus, lesion invasion, as well as age-related fatty infiltration and muscle atrophy. However, age exerts no impact on the contractility or fatigue resistance of the pectoralis muscle.^36^ In the present study, when placing ROIs in the pectoralis muscle as an internal reference, the slice with the greatest thickness of the pectoralis muscle was selected, and areas with visually evident fatty infiltration were carefully avoided to minimize the confounding effect of adipose tissue on the signal intensity of the muscle. Furthermore, for patients with tumor invasion of the pectoralis muscle, the contralateral pectoralis muscle was used as an internal control.

This study has the following limitations that need to be addressed. First, we used the signal value of the pectoralis muscle within the same patient as a reference for calculation, ignoring interpatient difference in pectoralis major muscle signal value, which might be a factor affecting T1 SIR and T2 SIR. Second, this was a single centre study with limited number of patients and unbalanced molecular subtypes. When measuring lesion size, only mass-like lesions were included, and lesions smaller than 5 mm were excluded, because excessively small lesions may compromise the accuracy of SIR. Future studies should include more patients and conduct multi-centre research to explore the combined application of MRI novel imaging technologies, and biomarkers. By extracting more refined imaging features, non-invasive assessment of intra-tumoral heterogeneity in breast cancer is expected to be achieved.

In conclusion, quantitative MRI parameters are correlated with the molecular subtypes of breast cancer. Combined parameters incorporating T1 SIR and T2 SIR exhibit potential diagnostic utility for differentiating HER2-enriched and triple-negative subtypes, respectively. T1 SIR and T2 SIR can enrich quantitative descriptors from breast MRI.
